# Health behaviours of young adults during the outbreak of the Covid-19 pandemic – a longitudinal study

**DOI:** 10.1186/s12889-021-11140-w

**Published:** 2021-06-02

**Authors:** Ewelina Czenczek- Lewandowska, Justyna Wyszyńska, Justyna Leszczak, Joanna Baran, Aneta Weres, Artur Mazur, Bogumił Lewandowski

**Affiliations:** 1 Institute of Health Sciences, Medical College, University of Rzeszów, ul. Kopisto 2a, 35-959 Rzeszów, Poland; 2 Institute of Medical Sciences, Medical College, University of Rzeszów, ul. Kopisto 2a, 35-959 Rzeszów, Poland

**Keywords:** Health-related behaviours, Young adults, Covid-19, Diet, Eating habits, Physical activity, Sedentary behaviours

## Abstract

**Background:**

The outbreak of the Covid-19 pandemic has created a number of obstacles to daily functioning, which have led to a need for major changes in lifestyles. The aim of the study was to assess whether and to what extent the outbreak of the Covid-19 pandemic has affected the health behaviours of young adults.

**Methods:**

506 people aged 18 to 34 ($\overline{x}$ =24.67 years ±4.23 years) who filled in an online survey were qualified for the study. Assessment was made of eating habits (a modified Food Frequency Questionnaire, FFQ), physical activity and sedentary behaviours (International Physical Activity Questionnaire, short form IPAQ-SF), sleep quality (The Pittsburgh Sleep Quality Index, PSQI), and generalized anxiety (Generalized anxiety Disorder, 7-item, GAD-7 scale).

**Results:**

During the pandemic, young adults changed their dietary preferences toward sweets and cereal products, increased alcohol (*p* < 0.001) and fat (*p* = 0.005) intake, significantly reduced their physical activity (from 8752.5 metabolic equivalents (MET) min/week vs. 6174.6 MET min/week, *p* < 0.001), increased the time spent sedentarily (Me = 240 min vs. Me = 360 min, p < 0.001) and had decreased sleep quality (PSQI score Me = 7.00 vs. Me = 9.00).

**Conclusions:**

The Covid-19 pandemic worsened health behaviours and intensified the feeling of generalized anxiety in young adults. Generalized anxiety during obligatory lockdown had the greatest negative impact on sedentary behaviours and sleep quality.

**Supplementary Information:**

The online version contains supplementary material available at 10.1186/s12889-021-11140-w.

## Background

Health-related behaviours include all activities that support, maintain or weaken the health and well-being of a human as a bio-psycho-social entity. There are two directions of behaviour, i.e. favourable (healthy behaviour) or threatening to health (unhealthy, self-destructive behaviour) [1]. Caring for physical health consists in maintaining personal hygiene and one’s own environment, undertaking appropriate physical activity as well as rational nutrition. Mental well-being is associated with the ability to cope with stress and maintain optimism, high self-esteem and a good perception of oneself and the environment. In turn, activity in the social sphere consists in building appropriate relationships and social communication, making new friends, participating in local organizations and associations. Participation in these spheres provides people with optimal well-being and prevents or delays the development of civilization diseases, e.g. myocardial infarction, stroke, diabetes, hypertension or obesity [2]. These diseases are causes of premature death and disability in old age, which is why shaping appropriate health attitudes and habits should apply to children, adolescents and young people.

Health-promoting behaviours are conditioned by many factors, both on the part of people and the environment. They can be habitual and result from deliberate and planned intentions, which are associated with appropriate knowledge and high levels of awareness of health issues. Reactive behaviour is a response to current social situations and requirements, such as emergencies, catastrophes or epidemics [3]. The outbreak of the SARS coronavirus infection represents a current and very strong environmental factor that has affected the lives of people around the world. This situation creates a number of difficulties in everyday functioning and requires the necessity to introduce changes in lifestyles. Despite these more difficult living conditions, it is especially important to take care of one’s own health during a developing pandemic.

A population worth separate analysis are young adults who, due to high educational and professional activity, are particularly vulnerable to experiencing major changes in their daily functioning. Young adulthood is a critical period of development between the time of adolescence and adulthood. So far, there are still no international guidelines for determining age ranges for young adults. The World Health Organization proposes a categorization of “young people” from 10 to 24 years of age, while some others define them as a group of people between 15 and 24 years of age, or even in some cases between 30 and 40 years of age [4]. So far, there is also a lack of an unambiguous characterisation of this group [5]. Rapid global economic and technological changes, prolonged transition to adult life, and delay in assuming work-related and family roles undoubtedly affect young adults’ health status. Due to increasing stress, sedentary lifestyles and unhealthy diet, today this population, despite their young age, is faced with a greater risk of obesity, hypertension, depression, oncological problems or serious mental health disorders. Furthermore, young adults appear to be less experienced in life than more mature people, who have already experienced other pandemics this century. These include the return of the influenza wave, caused by the mutant “Spanish” influenza virus (1918–1919) called H2N2 (1957–1958), followed by H3N2 (1968–1969) and H1N1 (1977, recurrence of the mutated version in 2009) [6]. The outbreak of the Covid-19 pandemic is therefore the first global event of this type encountered by the young population. This may be associated with lower psychological resistance and ability to cope with the stress related to the risk of falling ill.

The aim of the study was to assess whether and to what extent the outbreak of the Covid-19 pandemic influenced the health-promoting behaviours of young adults in terms of eating habits, physical activity, sedentary behaviours and sleep. The aim was also to assess the level of generalized anxiety and its impact on health-related behaviours of the studied group.

## Methods

### Study design and study sample

A retrospective-pre-post survey study was performed from 24 March 2020 to 11 April 2020 (a period of 19 days) using an online questionnaire survey method. The survey began 12 days after the pandemic was announced at the site of the study, i.e. between weeks 2 and 4 of obligatory lockdown, which entailed significant restriction of movement. Only participants in early adulthood (aged from 18 to 34 years) of Polish nationality resident in south-east Poland throughout the pandemic period were recruited in the study. The snowball sampling method was used. Questions in the online-survey were connected with health-behaviours in the pre-COVID-19 (recall) and COVID-19 periods. The pre-COVID-19 period was defined as the period 1 week before the mandatory quarantine, and the COVID-19 period as the period of 1 week before completion of the questionnaire. Participation in the study was voluntary and anonymous. At the beginning of the online survey, participants after reading all of the information about the study gave informed consent by clicking the option “Yes”. The study as well as the proposed method of obtaining consent were approved by the Ethics Committee of Rzeszów University (ref. no. 2/02/2019).

Data published by Statistical Office confirm that around 250,000 adults aged from 18 to 34 years live in south-eastern Poland. Assuming a confidence level of 95%, and a 5% margin of error, the required sample size should cover at least 384 participants. A completed survey was received from 690 respondents. Of those respondents, 184 were excluded from the study due to failure to meet the eligibility criteria (place of residence: south-eastern Poland, age from 18 to 34 years, lack of chronic comorbidities, lack of another health burden i.e. pregnancy, condition after surgery, musculoskeletal injury). The final study group consisted of 506 subjects.

### Measures

#### Food frequency questionnaire (FFQ)

To assess the habitual diets of the respondents, the modified Food Frequency Questionnaire (FFQ-6) was used [7]. The questionnaire included questions concerning the frequency of consumption of food products representing the main food groups: sweets and snacks; dairy products and eggs; grain products; fats; fruits; vegetables; meats; fishes; sweetened beverages; unsweetened drinks and alcohol. The respondents had a choice of five categories of food consumption frequency, with higher scores indicating more frequent food intake: (1) never or almost never, (2) 1–3 days /week, (3) more than 3 days / week (4) once a day, (5) several times a day. In the modified version the respondents provided two answers to each question, i.e. information on the last 7 days in the pandemic period and 7 days before the pandemic period.

#### Physical activity and sedentary behaviours (IPAQ – SF)

The levels of physical activity intensity and sitting time were measured using the 7-item International Physical Activity Questionnaire - Short Form (IPAQ – SF) [8]. The IPAQ - SF is one of the most widely used self-reporting questionnaires to assess physical activity [9]. The questionnaire consists of seven questions to capture the average daily time spent sitting, walking and engaging in moderate and vigorous physical activity over the last seven days.

The respondents provided two answers to each question, i.e. information on the last 7 days in the pandemic period and 7 days before the pandemic period. From IPAQ-SF, data were converted to the Metabolic Equivalent of Tasks (METs) minutes per week (MET-min/week) to gain an overall estimate of physical activity. One MET is defined as the energy expended while sitting quietly at rest and is equivalent to 3.5 ml/kg/min of VO2 Max [10].

#### The Pittsburgh sleep quality index (PSQI)

To measure sleep quality, The Pittsburgh Sleep Quality Index (PSQI) was used. The PSQI is an effective tool and the most widely used instrument used to measure the quality and patterns of sleep in different populations [11, 12]. The questionnaire has twenty-four items of which nineteen self-reported items are added non-linearly to generate seven components (subjective sleep quality; sleep latency; sleep duration; sleep efficiency; sleep disturbance; use of sleep medication and daytime dysfunction). The original version of the questions concerns the period of the last month, while the authors asked the respondents about the last 7 days during the pandemic period and 7 days before the pandemic period. The scores of these components are pooled to obtain a global PSQI score, which is a measure of sleep health (0–21 points). Higher scores represent worse sleep quality.

#### Generalized anxiety disorder 7-item (GAD-7 scale)

For screening Generalized Anxiety Disorder (GAD) and assessing its severity, the Generalized Anxiety Disorder 7-item (GAD-7) questionnaire was used. The GAD-7 is a self-administered, valid and efficient tool used in clinical practice and research [13]. The GAD-7 has shown good reliability and construct validity [14]. The questionnaire represents an anxiety measure based on seven items which are scored from zero to three (feeling nervous, anxious, or on edge; being able to stop or control worrying; worrying too much about different things; trouble relaxing; being restless; becoming easily annoyed or irritable; and feeling afraid as if something awful might happen). The whole scale ranges from 0 to 21, with higher scores reflecting greater severity of anxiety. The cut-off scores for minimal, mild, moderate and severe anxiety symptoms are 0–4, 5–9, 10–14 and 15–21, respectively [15]. The respondents provided two answers to each question, i.e. information on the last 7 days in the pandemic period and 7 days before the pandemic period.

#### Socio-demographic and socioeconomic data, authors’ own questionnaire

Socio-demographic and socioeconomic characteristics (such as age, gender, education, employment status, housing conditions, place of residence) were self-reported by the respondents in the first part of the questionnaire [Additional File 1].

### Statistical analysis

Data analysis was performed using Statistica 13.1 statistical software. A non-parametric test – the Wilcoxon signed-rank test – was used to compare intra-group variability in the period before and during lockdown. The Spearman’s rank correlation coefficient was used to assess the relationship between the size of the changes and selected factors. The level of statistical significance was adopted at *p* < 0.05.

## Results

From a group of 690 people surveyed, a total of 506 people aged 18 to 34 ($\overline{x}$ =24.67 years ±4.23 years) met the inclusion criteria. The study group included 355 females (70.2%) and 151 males (29.8%). Most of the respondents, i.e. 361 people (71.3%) had normal body weight, 26 people (5.1%) were underweight, 90 (17.8%) overweight and 29 obese (5.7%).

Most of the people lived in larger houses > 100 m2 (49.2%) or smaller houses < 100 m2 (24.3%). To a similar degree, the respondents came from rural (45.5%) and urban (54.6%) environments of varying sizes from ten thousand inhabitants up to a million inhabitants. Similar numbers of respondents had secondary (52%) and higher (46%) education. The largest groups of respondents were students (36.8%) or were professionally inactive during the pandemic (23.7%). Some of the respondents (20%) had not changed the way they worked and were professionally active on the same basis as before the pandemic period, and 13% had changed their way of working to remote work performed at home during the pandemic period. Detailed data on the socio-demographic characteristics of the study group are provided in Table 1 (Table 1).

**Table 1:** Sociodemographic characteristics of the respondents

Variable	N	%	$\overline{x}$ +/− SD
**Age (years)**	506	100	24.67 +/− 4.23
**Gender**			
Female	355	70.2	
vMale	151	29.8	
**BMI Category**			
Normal	361	71.3	
Underweight	26	5.1	
Overweight	90	17.8	
Obese	29	5.5	
**Living conditions**			
Flat in a block < 50 m2	52	10.3	
Flat in a block 50–100 m2	72	14.2	
Flat in a block > 100 m2	10	2.0	
House 50–100 m2	123	24.3	
House > 100 m2	249	49.2	
**Place of residence**			
Countryside	230	45.5	
Town< 10 thousand residents	56	11.1	
Town 10–100 thousand residents	96	19.0	
City 100–300 thousand residents	95	19.0	
City 300 thousand-1 million residents	28	5.5	
City > 1 million residents	1	0.2	
**Education**			
Secondary	263	52.0	
Basic vocational	10	2.0	
Higher	233	46.0	
**Employment status during the pandemic**			
Unemployed	33	6.5	
Professionally active as before	101	20.00	
Remote work during the pandemic	66	13.00	
Not professionally active during the pandemic	120	23.7	
Student/pupil	186	36.8	
**Smoking**			
No	390	77.1	
From time to time	68	13.4	
Yes, 0–5 years	28	5.5	
Yes, 5–10 years	13	2.6	
Yes, > 10 years	7	1.4	

N- number of participants; $\overline{x}$- average, SD- standard deviation.

Changes in the average consumption of selected food products in the periods before and during the pandemic were recorded as statistically significant for: sweets and snacks (*p* = 0.043), cereal products (*p* = 0.011), fats (*p* = 0.005) and for alcohol (*p* < 0.001).

In the case of sweets and snacks and cereal products, the changes were not visible in the whole group, i.e. the median and quartile ranges were the same before and during the pandemic. Hence, the significance of the differences in this case pointed to intra-group changes. People who used to eat fewer sweets or cereal products ate more during the pandemic, while others who previously consumed more of them reduced their consumption. It can be concluded that individual preferences have changed, but looking at the group globally, consumption was similar in the period before and during the pandemic (only the population breakdown changed). In the case of alcohol and fats, a significant increase in their consumption was noted in the whole group in general (*p* < 0.001, *p* = 0.005) (Table 2).

**Table 2:** Average consumption of selected food products before and during the pandemic

Products scale of consumption 1 – never / almost never 2–1-3 days/week 3 - > 3 days/week 4–1 time/day 5 – several times a day	Period of 7 days before pandemic			Period of 7 days during pandemic			P
	Me	Q1	Q3	Me	Q1	Q3	
Sweets and snacks	3.0	2.0	4.0	3.0	2.0	4.0	0.043*
Dairy products and eggs	4.0	2.0	4.0	4.0	3.0	4.0	0.179
Cereal products	4.0	3.0	4.0	4.0	3.0	4.0	0.011*
Fats	3.0	2.0	4.0	4.0	2.0	4.0	0.005*
Fruit	4.0	2.0	4.0	4.0	2.0	4.0	0.433
Vegetables and wholegrains	3.0	2.0	4.0	3.0	2.0	4.0	0.058
Meat products	3.0	2.0	4.0	3.0	2.0	4.0	0.390
Fish	2.0	1.0	2.0	2.0	1.0	2.0	0.817
Sweetened drinks	2.0	1.0	2.0	2.0	1.0	2.0	0.722
Unsweetened drinks	2.0	1.0	2.0	2.0	1.0	3.0	0.682
Alcohol	1.0	1.0	2.0	2.0	1.0	2.0	< 0.001*

P- probability level test; Me- median value; Q1- Quartile 1; Q3- Quartile 3; *- statistical significance at p < 0.05.

The overall level of activity of the subjects during the pandemic period significantly decreased in comparison to the period before the pandemic (*p* < 0.001). The average value (Me) of the level of physical activity (IPAQ) before the pandemic was 8752.5 MET min/week, while during the pandemic it dropped to 6174.6 MET min/week.

The time spent in a sitting position increased statistically significantly (sedentary behaviour increased) during the pandemic compared to the period before the pandemic (*p* < 0.001). The average value (Me) of sitting time before the pandemic was 240 min, while during the pandemic it was 300 min.

On the PSQI scale, there was a statistically significant decrease in the number of points obtained during the pandemic compared to the period before the pandemic (*p* < 0.001), which indicates a significant decrease in sleep quality. The average overall PSQI score (Me) before the pandemic was 7.00, and during the pandemic it increased to 9.00. Detailed data is presented in Table 3 (Table 3).

**Table 3:** General indicators of physical activity, sedentary behaviour and sleep quality in the pre-pandemic period and during the pandemic

	Descriptive statistics							
TOTAL MET minutes of physical activity a week [MET min./week.]	Physical activity (IPAQ-SF)							
	N	$\overline{x}$	Me	Min.	Max.	Q1	Q3	SD
Before pandemic	506	8876.8	8752.5	0.0	46,572.0	5403.0	11,820.0	5288.9
During pandemic	506	6174.6	5483.0	0.0	28,314.0	2380.0	9009.0	4933.3
Difference	506	− 2702.1	− 1980.0	−21,849.0	16,638.0	− 5548.5	0.0	4497.1
P	Z = 12.39 p < 0.001							
TOTAL minutes of sitting position a week [minutes]	Sedentary behaviours (IPAQ-SF)							
	N	$\overline{x}$	Me	Min.	Max.	Q1	Q3	SD
Before pandemic	506	262.2	240.0	0.0	720.0	120.0	360.0	162.6
During pandemic	506	309.0	300.0	0.0	960.0	180.0	420.0	172.9
Difference	506	46.7	40.0	− 580.0	480.0	0.0	150.0	156.0
P	Z = 6.97 p < 0.001							
	Sleep quality (PSQI)							
Sleep qualityPSQI - Total score	N	$\overline{x}$	Me	Min.	Max.	Q1	Q3	SD
Before pandemic	506	7.25	7.00	0.00	15.00	5.00	9.00	2.63
During pandemic	506	8.82	9.00	1.00	18.00	6.00	11.00	3.38
Difference	506	1.58	1.00	−6.00	12.00	0.00	3.00	2.37
P	Z = 12.64 p < 0.001							

N- number of participants; $\overline{x}$- average, Me- median value; Min.- minimum; Max.- maximum; Q1- Quartile 1; Q3- Quartile 3; SD- standard deviation; Z- Wilcoxon matched-pairs signed rank test result; P- probability level test; PSQI Total score- higher scores represent worse sleep quality.

Analysing the results of the generalized anxiety level, its statistically significant deterioration (*p* < 0.001) during the pandemic was confirmed (Table 4).

**Table 4:** The level of generalized anxiety before the pandemic and during the pandemic

Level of generalized anxiety (GAD 7)	Period of 7 days before pandemic		Period of 7 days during pandemic	
	N	%	N	%
Mild	378	74.7%	243	48.0%
Moderate	93	18.4%	166	32.8%
Moderately severe	26	5.1%	57	11.3%
Severe	9	1.8%	40	7.9%
Total	506	100.0%	506	100.0%
Significance (p)	Z = 9.59 p < 0.001			

N- number of participants; Z- Wilcoxon matched-pairs signed rank test result; P- probability level test.

The younger the subjects were, the more their level of physical activity was reduced, and the more frequent their sedentary behaviour became. During the pandemic female participants were less physically active and spent more time sedentarily. People with a lower level of education experienced greater intensification of generalized anxiety than better educated people. The more people smoked, the less noticeable was the deterioration in sleep quality. Sleep quality worsened in non-smokers and those who smoke less often. People who experienced more generalized anxiety during the pandemic showed a greater change in the time spent sedentarily, i.e. they were sitting much longer than those who experienced less generalized anxiety. People experiencing greater generalized anxiety during the pandemic had a greater reduction in sleep quality, as well as a more noticeable change in perceived generalized anxiety relative to the pre-pandemic period (Table 5).

**Table 5:** The size of changes in the variables studied versus age, gender, BMI category, living conditions, place of residence, education, smoking and level of generalized anxiety during the pandemic

Variables		Physical activity	Sedentary behaviours	Sleep quality	Generalized anxiety
Age	R	0.13	−0.13	− 0.00	0.04
	P	0.005*	0.003*	0.958	0.359
Gender	R	−0.09	0.008	0.008	− 0.03
	P	0.042*	0.081	0.086	0.456
BMI Category	R	0.03	0.03	0.07	0.07
	P	0.565	0.537	0.111	0.119
Living conditions	R	0.07	−0.06	−0.03	− 0.04
	P	0.128	0.203	0.455	0.373
Place of residence	R	0.03	0.01	−0.07	−0.08
	P	0.517	0.779	0.131	0.067
Education	R	0.07	−0.01	−0.04	−0.15
	P	0.123	0.782	0.369	0.001*
Smoking	R	−0.07	−0.01	0.10	0.01
	P	0.134	0.884	0.026*	0.860
Generalized anxiety during pandemic	R	−0.04	0.09	−0.28	0.73
	P	0.350	0.046*	0.001*	0.001*

R- Spearman rank correlation; P- probability level test; *- statistical significance at p < 0.05.

## Discussion

Caring for mental and physical well-being during the Covid-19 pandemic is a significant global issue and it affects people around the world. Like all other epidemics to date, the Covid-19 pandemic and its associated restrictions (including strict quarantine, limitation of social activity) carry an increased risk of negative psychological reactions. First of all, these include symptoms of high anxiety, depression, stress and feelings of loneliness [16]. For the first time, young adults have encountered the phenomenon of a dangerous global pandemic that threatens health and life. To date, most specialists have focussed on the elderly who are most exposed to the negative effects of the pandemic, which is why we attempted to analyse the magnitude of the effects of the pandemic in the young population [17, 18].

### Eating habits

Another important pillar of human health is rational nutrition based on nutritious food products, full of vitamins (e.g. A, C, D and E) and trace elements (e.g. zinc, selenium, iron), which support the immune system and many other physiological processes [19]. A period of isolation is associated with high psychological pressure. This situation carries the risk of people eating larger amounts of food at a higher frequency, as well as changing the preferred products in their diet [20]. This is confirmed by Zachary’s results, in which as many as 52% of respondents increased their food intake in response to stress, and 73% ate when they felt bored during quarantine [21]. This thesis was also confirmed by our own results, as the respondents changed some food preferences, e.g. in the case of sweets and cereal products. Various directions of changes were observed (both increasing and decreasing), but when analysing the group as a whole, consumption was similar in both periods. It was different in the case of alcohol and fats, where a significant increase in their consumption has been confirmed. Similarly, Flaudias observed that stress related to the lockdown and social isolation increased the risk of problematic eating behaviours among young people e.g. binge eating disorder and dietary restriction [22]. The international ECLB-COVID19 electronic survey confirms that food consumption and meal patterns were more unhealthy during home confinement, but in contrast with our own study, alcohol consumption decreased significantly [23]. Rehm draws attention to two potential main mechanisms of the possible impact of Covid-19 on alcohol use. The first is the model of increased alcohol consumption, mainly among men, associated with greater stress, loss of work and a change in the lifestyle that had been pursued so far. Some studies show up to six times higher alcohol consumption during the pandemic among male than female respondents [24]. The second model assumes reduced alcohol consumption due to reductions in income and the closing of on-premise consumption sites [25].

Abbas emphasizes the problem of widespread obesity during the Covid-19 pandemic, which results, among others, from lower physical activity during restrictions and consumption of more energy-dense foods that are rich in fat and sugar. Although no relationship between health behaviours and BMI was found in our study, similar behavioural patterns were observed. Increased stress associated with the pandemic adversely affects the brain areas responsible for the ability to self-regulate that are needed to control health behaviours, including nutrition and physical activity, which are the basis for controlling weight. Stress reduces the desire for physical activity, strengthens the tendency to eat more food and disturbs sleep by shortening its length. All these factors contribute to the development of obesity, which has been a global problem for many years, including among the populations of children, adolescents and young adults [26].

### Physical activity (PA) and sedentary behaviour (SB)

One of the key health-promoting behaviours that improves physical fitness but also supports mental health is physical activity (PA) and avoidance of sedentary behaviour (SB) [27]. From the point of view of the current epidemiological situation, PA is even more important than before, because in addition to improving physical and mental condition, it stimulates immune mechanisms [28]. Exercise has been shown to reduce anxiety and depression symptoms, as well as improve self-esteem and cognitive function [29]. Huang’s extensive meta-analysis confirms that mentally passive SBs increase the risk of depression [30].

Just 150 min of moderate-to-vigorous intensity aerobic physical activity per week (MVPA) or a minimum of 10,000 steps/day, bring positive benefits to young adults’ health, and these recommendations sill apply during the pandemic [31, 32]. The initial period of the pandemic significantly reduced the frequency with which people left their homes and the time they spent in recreational places, such as swimming pools, gyms, fitness clubs, parks and playgrounds, making it significantly more difficult for them to be active. The Covid-19 pandemic has promoted sedentary behaviour, as the possibilities of being active are limited and, if they are available at all, they are associated with fear of infection and an attitude of withdrawal. In this context, the pandemic creates highly adverse conditions with a risk of long-term consequences [33]. The results of our own study confirmed a significant decrease in PA and a simultaneous increase in SB among young adults. In the young adult population of the south-eastern region of Poland, adverse changes were found particularly among younger people and females. Using the overall IPAQ indicator, there was a significant decrease from 8752.5 MET min/week (pre-pandemic period) to 6174.6 MET min/week (during the pandemic). In the case of the time declared to have been spent on SB, a significant increase was recorded from 240 min (4 h) to 300 min (5 h). Zachary obtained results from an online survey method, which shows that adults during quarantine spent an average of 4.8 h per day watching TV or playing video games and 2.5 h per day on a computer (in total about 7.3 h per day of SB) and that they accumulate 2.7 h per week of PA [21]. The international ECLB-COVID19 electronic survey also confirmed that home confinement had a negative effect on all PA intensity levels (vigorous, moderate, walking and overall), and daily sitting time increased from 5 to as much as 8 h per day [22].

Currently, little is known about the effect of PA on the susceptibility to SARS-CoV-2 infection. However, it is emphasized that disruption of daily exercise routines and reduction of the current daily PA may increase predisposition to viral infections and comorbidities, i.e. heart disease, type 2 diabetes, obesity and hypertension, which significantly worsen the course of a Covid-19 infection [34]. PA is recommended in the fight against the negative effects of the pandemic at every stage, including in the future, because it enhances the efficacy of vaccines [35, 36]. It is recommended that during the pandemic people of all ages try to be as active as possible, and the safest form is moderate intensity exercise performed at home, using applications or online videos with instructors [37–39].

### Sleep quality

Hygiene and the quality of sleep are important elements of everyday functioning, and so special attention is paid to them in modern times. It turns out that providing the right amount of sleep and stable sleeping times are necessary for the smooth functioning of the immune system and circadian hormonal rhythm, which are extremely important during a pandemic. Using the PSQI scale, the study group noted a significant decrease in sleep quality during the pandemic and an increase in the value of the PSQI global score from 7.00 to 9.00. Casagrande reports that the group she studied had lower sleep quality during the pandemic, where the PSQI global score was 5.69, compared to general data from Curcio of 4.00. Female subjects had a higher risk of sleep disorders. The results of our own study obtained higher results than both of those authors, therefore the sleep quality of the studied group was even lower [40, 41]. Romero-Blanco observed that the worsening of sleep quality occurred mainly in the subgroups: female, first-year students and second-year students [42]. Luciano pointed out that hours sitting per day was higher among young adults who were sleeping less than 7 h per night during the pandemic [43].

The results for smokers are debatable. The more the people surveyed smoked, the less noticeable was their deterioration in sleep quality, as opposed to non-smokers and those who smoke less often, who more often showed worse sleep. This result can be explained by the fact that despite the fact that smoking negatively affects health, during the pandemic it played an anti-stress role, which was associated with relatively better quality of sleep. Different results were obtained by Liao, where the PSQI global score for smokers was 4.72 and for non-smokers 4.11, which indicated worse quality of smokers’ sleep [44]. The contradictory research results should be explained by the completely different conditions caused by the pandemic and altered human responses during this period. In the Liao study, people from all age groups participated, starting from adolescents 12–17 years old, where as many as 6.6% of this group smoked, while in the age group 18 to 29 years old - 36.9%. These rates were much higher than in our own study, where there were 9.5% regular smokers among the respondents. Patterson reports that sleep disorders are often seen in people who have given up smoking, as many as 43% of whom report insomnia, and even up to 80% report some kind of sleep disorder, thus confirming the strong relationship between cigarette smoking and sleep quality [45].

### Generalized anxiety and health-promoting behaviours

The work also addresses an important aspect of the feeling of generalized anxiety among young adults, which increased significantly during the pandemic, especially in people with lower level of education. In the study conducted by Ahmed, as many as one-third of the respondents (37.6%) reported lower perceived mental well-being, and most was reported by young people between 21 and 30 years of age. Similarly, in the case of having different levels of anxiety and depression, it was young people who more often had problems with their mental state than older people [20]. In our own study, people who experienced higher generalized anxiety spent much more time sedentarily, i.e. they were sitting for much longer, primarily watching TV and browsing the Internet on a tablet or a mobile phone. Specialist recommendations seek to limit the viewing of current news about the pandemic on TV, which, due to the huge amount of overwhelming information, adversely affects people’s mental state. As confirmed by our own research, this also had an impact, worsening the quality of sleep, especially for people who experience high generalized anxiety. In our study, the overall score on the GAD 7 scale in the pre-pandemic period was 3.63 points, while during the pandemic it increased to 6.70 points, which indicates a worsening of symptoms. In the Casagrande study, the generalized anxiety of respondents during the pandemic was 7.61 on the GAD 7 scale, and was significantly higher compared to general data from Löwe, which was 2.95. Although the respondents came from different age groups, it was judged that females between 18 and 30 years of age were most at risk of anxiety. This confirms that young adults are a vulnerable group that suffered many negative effects of the pandemic [15, 41].

### Summary

In recent decades, modern health behaviours of young adults have largely departed from the adopted recommendations. The presence of the Covid-19 pandemic reinforces abnormal health behaviour patterns that are already common among young people and significantly increases their anxiety. Orders to prevent the spread of Covid-19 disease create even more difficult conditions for the improvement of health-related behaviours among the young population. It should be taken into account that poor health habits, both in the context of physical and mental health, may aggravate other existing public health problems. The young population conducting unhealthy lifestyles is linked not only with a higher risk of non-communicable disease (NCD) but also of Covid-19 hospital admission [46]. That is why it is so important in this particular period to emphasize even more the importance of shaping people’s health behaviours, with particular attention being paid to the young population.

### Limitations

The presented study was completed shortly after the beginning of the outbreak of the pandemic and comes from only one region of Poland. One of the limitations was the inability to use objective research methods, requiring direct contact with the subject, during an ongoing pandemic. However, the study used the most common and standardized questionnaires, which are recognized survey methods. The presented data describe the subjective feelings of the respondents, and information from before and during the pandemic period is obtained using the recall method. The study may go further to cover other important threads of health behaviour during the pandemic, i.e. a thorough analysis of smoking or oral health, dental check-ups, medical screenings or risky driving.

## Conclusions

Young adults during the pandemic significantly reduced their levels of physical activity, increased the time spent sedentarily, had worse sleep quality, changed their eating preferences regarding the consumption of sweets and cereal products and significantly increased their consumption of alcohol and fats. During the pandemic, there was a significant increase in the feeling of generalized anxiety in young adults, which had an impact on the deterioration of sleep quality and prolonged the time spent sedentarily.

## Supplementary Information

**Additional file 1.** Authors’ own questionnaire. The file contains the content of the questions from the authors’ own questionnaire (part I), as well as the name and sources of the standardized questionnaires used (part II).

## Data Availability

The data are available on reasonable request.

## Abbreviations

FFQ: Food Frequency Questionnaire
IPAQ-SF: International Physical Activity Questionnaire
PSQI: The Pittsburgh Sleep Quality Index
GAD-7 scale: Generalized anxiety Disorder
MET: metabolic equivalents
PA: physical activity
SB: sedentary behaviour
