# Supplementary material for: Health behaviours of young adults during the outbreak of the Covid-19 pandemic – a longitudinal study
Source: BMC Public Health. 2021 Jun 2;21:1038. doi: 10.1186/s12889-021-11140-w (PMC8170456; doi:10.1186/s12889-021-11140-w)
Supplement: Supplementary file 1 — Additional file 1. Authors’ own questionnaire. The file contains the content of the questions from the authors’ own questionnaire (part I), as well as the name and sources of the standardized questionnaires used (part II). [file 12889_2021_11140_MOESM1_ESM.pdf]

Author's own questionnaire

Dear Respondens,

We would like to ask you to voluntarily and anonymously fill out onine quesstionnaire as a part of scietific research from the University of Rzeszow. The survey concers about your health-related habits during the outbreak of Covid-19 pandemic. The questions come from author's own questionnaire as well as standardized international questionnairies related to eating habits, physical activity, sleep quality and mental well-being.

Please state that You have read all of the information about the study and that you give a consent to the participation (obligatory field):

☐ **YES** ☐ **NO**

Thank You in the participation in the study.

PART I (author's own questionnaire) **Socio-demographic information**

1. **Sex** ☐ woman ☐ man
2. **Age** …………………………..
3. **Weight (kg)** ……………………….. **Height (cm)** …………………………..
4. **Place of residence**

☐ Countryside
☐ Town< 10 thousand residends
☐ Town 10-100 thousand residends
☐ City 100-300 thousand residends
☐ City 300 thousand- 1 milion residents
☐ City > 1 million residends

5. **Living conditions**

☐ Flat in a block < 50m2
☐ Flat in a block 50- 100 m2
☐ Flat in a block > 100m2
☐ House 50- 100 m2
☐ House> 100m2

6. **Number of house members** number of adults……..….number of children ………………..
7. **Education** ☐ Secondary ☐ Basic vocational ☐ Higher
8. **Employment status during the pandemic**

☐ Unemloyed professionally active as before
☐ Remote work during the pandemic
☐ Student/pupil

9. **Type of the work** ……………………..
10. **Comorbidities** ……………………………………………………………………

11. Smoking

- ☐ yes, 0-5 years
- ☐ yes, 5-10 years
- ☐ yes, >10 years
- ☐ no
- ☐ from time to time

12. **Additional information, my status of COVID-19 during the study**
…………………………………..

PART II

- FFQ6- modified version (respondents provided two answers to each question, i.e. information on the last 7 days in the pandemic period and 7 days before the pandemic period. In orginal version questions concern about 12 months ago).

Wądołowska L. Validation of food frequency questionnaire [FFQ]. Reproducibility assessment. Bromatol Chem Toksyk. 2005;38,1:27-33.

-IPAQ-SF – short version (orginal)

Biernat E, Stupnicki R, Gajewski AK. Międzynarodowy Kwestionariusz Aktywności Fizycznej (IPAQ) – wersja polska. Wychowanie Fizyczne i Sport. 2007;51,1:47-54.

- Pittsburgh Sleep Quality Index (PSQI) questionnaire(orginal)

Buysse DJ, Reynolds CF, Monk TH, Berman SR, Kupfer DJ. The Pittsburgh Sleep Quality Index: a new instrument for psychiatric practice and research. Psychiatry Res. 1989;28:193–213.

GAD-7 Anxiety Severity (orginal)

Spitzer RL, Kroenke K, Williams JBW, Lowe B. A brief measure for assessing generalized anxiety disorder. Arch Inern Med. 2006;166:1092-1097.
